# Dr. Chester Howard

**Published:** 1903-07

**Authors:** 


Dr. Chester Howard, of Dayton, N. Y., University of Buffalo, 1879, died at his home, May 27, 1903, aged 55 years. He had been an invalid for two years or more from an affection of the spine.

